# Anti-Interleukin-6 Receptor Antibody Therapy-Induced Retinopathy in a Patient with Rheumatoid Arthritis

**DOI:** 10.1155/2012/270315

**Published:** 2012-12-24

**Authors:** Asumi Tada, Noriyasu Hashida, Toshio Tanaka, Kohji Nishida

**Affiliations:** ^1^Department of Ophthalmology, Osaka University Graduate School of Medicine, E7, 2-2 Yamadaoka, Suita, Osaka 565-0871, Japan; ^2^Department of Respiratory Medicine, Allergy and Rheumatic Diseases, Osaka University Graduate School of Medicine, E7, 2-2 Yamadaoka, Suita, Osaka 565-0871, Japan

## Abstract

Tocilizumab, a humanized anti-human interleukin-6 (IL-6) receptor monoclonal antibody, is beneficial for treating autoimmune conditions such as rheumatoid arthritis (RA). The most common adverse event is upper respiratory tract infection; ocular side effects are rare. We describe a case of skin ulceration and bilateral retinopathy with multifocal cotton-wool spots and retinal hemorrhages in a patient with RA treated with tocilizumab. Tocilizumab administration increased the serum level of IL-6 without affecting the IL-8 levels. We could not exclude the possibility of blood coagulation or retinal vascular changes caused by tocilizumab. The current case highlights the need to consider that ocular adverse effects can develop in patients treated with tocilizumab.

## 1. Introduction

Interleukin-6 (IL-6) is an immunoregulatory cytokine that plays an important role in autoimmunity. Signaling abnormalities in IL-6 could lead to an autoimmune disease or increased susceptibility to infection [1]. Tocilizumab (Actemra, Chugai Pharmaceutical Co., Ltd., Tokyo, Japan), a humanized anti-human IL-6 receptor (IL-6R) monoclonal antibody that inhibits IL-6 binding to IL-6R might inhibit the biologic function of IL-6, which plays a pivotal role in the pathogenesis of autoimmune diseases. Recent reports have indicated that tocilizumab also may be beneficial for treating autoimmune diseases such as rheumatoid arthritis (RA), Castleman's disease, and juvenile idiopathic arthritis [1–4]. The most common adverse events associated with the drug are upper respiratory tract infections; however, some serious adverse events have been reported previously [2–5] and ocular side effects are especially rare [6, 7]. We report the case of a patient with RA who was treated with tocilizumab and developed bilateral retinopathy with multiple cotton-wool spots and bilateral retinal hemorrhages.

## 2. Case Report

The patient was a 43-year-old woman who had been diagnosed with RA 9 years previously. Before tocilizumab was administered intravenously, she had been treated with an 8-mg weekly dose of methotrexate, and the disease activity was well controlled. However, after 15 months of treatment, the RA was refractory to treatment and she was referred to our hospital. At baseline, the disease activity score in 28 (DAS28) joints was 2.39. She had no complications such as diabetes, hypertension, or systemic viral infections. Routine ophthalmologic examinations, including measurement of the best-corrected visual acuity (BCVA) and intraocular pressure, slit-lamp examination, and fundus examination, were unremarkable.

**Figure 1:**
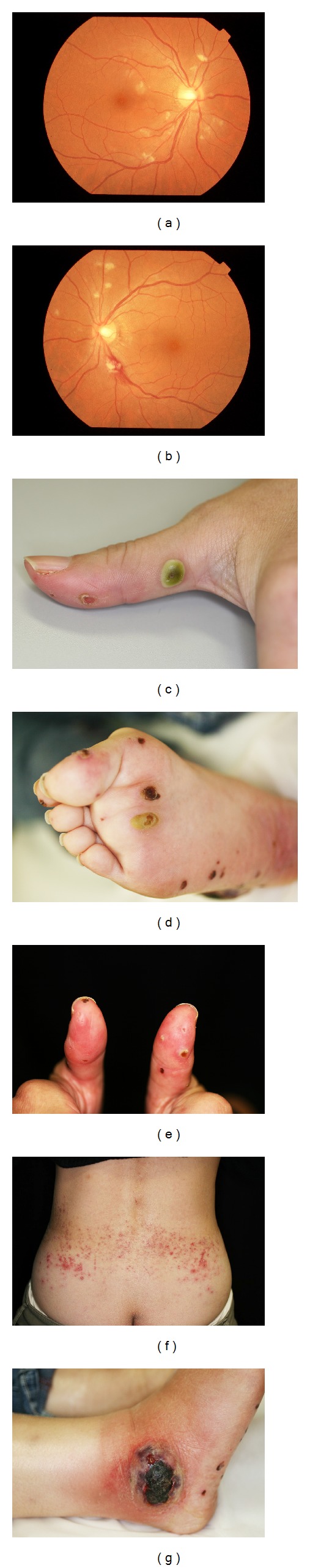
Adverse events after tocilizumab therapy. (a), (b) Photographs show the bilateral fundus images of retinopathy with bilateral multiple cotton-wool spots and retinal hemorrhages around the optic disc. (c), (d), and (e) Rashes on the palms and planta pedis resemble pustulosis palmoplantaris, and (f) diffuse pruritic erythema around the lumbar region appeared 20 days after the first infusion of tocilizumab. (g) Skin ulceration on the right external malleolus appeared immediately after the second infusion of tocilizumab.

The patient received 8 mg/kg tocilizumab intravenously while she was in the hospital.  Table 1 shows her laboratory data on the day of treatment. Twenty days after treatment, rashes inexplicably developed on the palms and planta pedis that resembled pustulosis palmoplantaris, stomatitis on the tongue, and diffuse pruritic erythema around the lumbar region (Figures 1(c)–1(f)). The skin lesions showed no signs of infection, and tocilizumab was continued. Immediately after a second infusion, a skin ulcer developed on the right external malleolus (Figure 1(g)). Four weeks after the second tocilizumab administration, this area became necrotic. She was treated with alprostadil alfadex (Prostandin, Ono Pharmaceutical Co., Ltd., Osaka, Japan) and iodine ointment after the necrotic tissue was debrided. Methicillin-susceptible *Staphylococcus aureus* was cultured from the lesion.

Laboratory analyses of blood samples showed no abnormalities except an elevated serum C-reactive protein value (3.01 mg/dL). A chest X-ray and chest computed tomography images were normal. The tuberculin skin test and the QuantiFERON test (Cellestis Limited, Carnegie, VIC, Australia) were negative. There was no sign of infectious endocarditis on cardiac ultrasonography. A biopsy of the skin ulcers 2 months after tocilizumab administration showed no apparent vasculitis, microangiopathy, or embolus. The joint swelling and tenderness worsened and the DAS28 score 2 months after tocilizumab treatment increased to 5.69. At this time, the BCVA was 20/15 bilaterally. No significant inflammation was seen in the anterior segment. No inflammatory cells in the vitreous or vasculitis were seen; however, the fundus examination showed multiple cotton-wool spots and retinal hemorrhages surrounding the optic disc bilaterally (Figures 1(a) and 1(b)).

**Figure 2:**
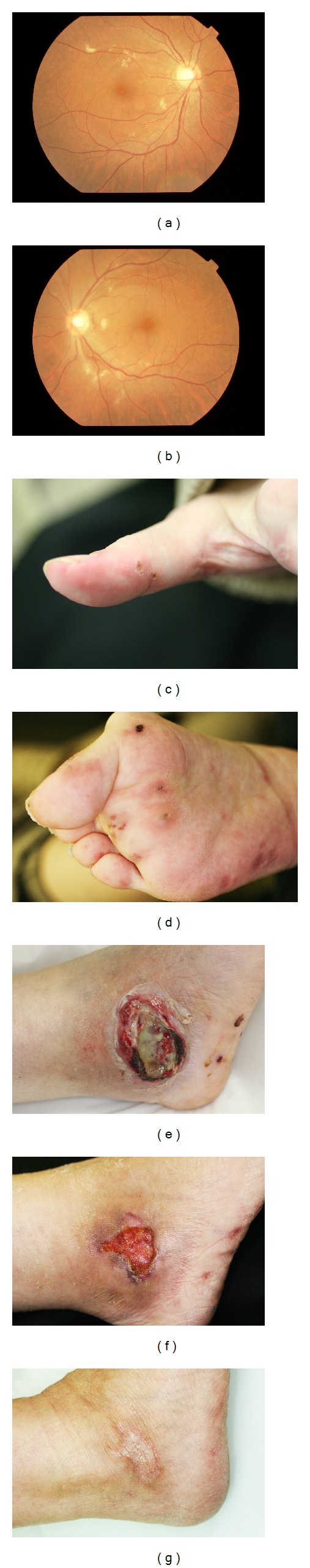
Four months after tocilizumab was discontinued and antibiotic therapy was started, (a, b) ocular findings and (c, d) rashes on the palm and planta pedis are improving gradually. The ulcerative infected skin lesions gradually resolved (e), (f), and (g). (e) Two days after debridement. The skin lesions resolved (f) 4 and (g) 9 months after discontinuation of tocilizumab.

**Figure 3:**
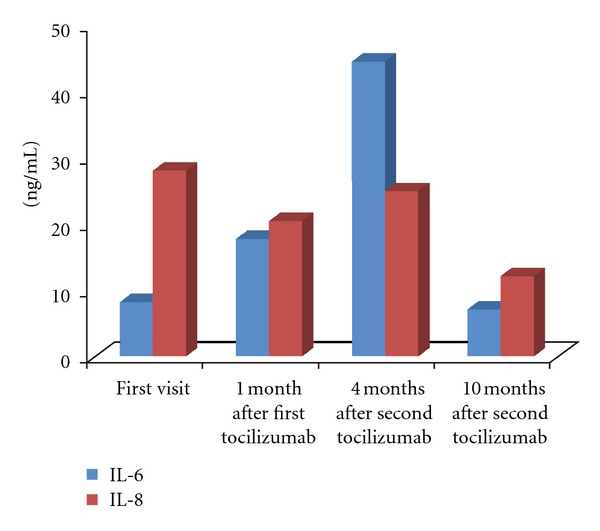
Serum concentrations of IL-6 and IL-8 before and after tocilizumab administration. Marked increases in the serum levels of IL-6 are observed 1 and 4 months after tocilizumab administration that did not affect the serum levels of IL-8.

After tocilizumab was discontinued, antibiotic therapy (systemic cefcapene pivoxil hydrochloride hydrate (300 mg three times daily) for 3 weeks) and oral prednisolone (up to 30 mg/day) gradually ameliorated the necrotic tissue formation on the skin ulcers (Figures 2(c)–2(g)). The prednisolone was tapered by 5 mg/month to 5 mg/day. The ocular findings improved concurrently with resolution of the skin lesions; the cotton-wool spots remained, but the retinal hemorrhages gradually resolved 4 months after treatment (Figures 2(a) and 2(b)). The patient's disease activity improved and skin ulceration showed complete epithelialization (Figure 2(g)). Her DAS28 score 3 months after tocilizumab administration decreased to 0.21. We also examined the serum levels of IL-6 and IL-8 before and after tocilizumab administration. The changes in these molecules are shown in Figure 3. We found markedly increased serum levels of IL-6 at 1 and 4 months after tocilizumab administration that did not affect the serum levels of IL-8.

## 3. Discussion

Tocilizumab has anti-inflammatory properties by inhibiting IL-6 signaling, and the drug is beneficial for treating autoimmune conditions such as RA. The most common adverse event reported has been mild upper respiratory tract infection such as nasopharyngitis. We described, for the first time, fundus manifestations associated with tocilizumab infusions, that is, bilateral retinopathy with multifocal cotton-wool spots and retinal hemorrhages. Concerning the vasculitis caused by biologics, previous investigators have reported that vasculitis can occur after administration of antitumor necrosis factor-*α* antibody [8, 9]. IL-6 exacerbates blood coagulation. As a result, binding inhibition of IL-6 with IL-6R via tocilizumab administration causes an excessive increase of IL-6, suggesting that peripheral thrombosis might result [5]. The excessive increase of serum IL-6 was confirmed by the active fundus changes in the current case. Therefore, fundus and histologic examinations revealed no vascular changes; however, we could not exclude the possibility of blood coagulation or immune complex mediated vasculitis in this patient. 

The occurrence of ocular adverse events associated with tocilizumab therapy indicates involvement of the infectious skin ulcers and direct involvement of tocilizumab, or both. We have to consider both possibilities. A possible explanation for the infection is that there was no evidence of vasculitis in the skin biopsy and ocular examinations. The ocular findings markedly improved concurrently with resolution of the skin lesions with antibiotic therapy. This possibility is supported by a report of ocular findings that were similar to the ocular manifestations of infectious endocarditis [10]. Regarding the direct involvement of tocilizumab, the drug itself might directly affect the retinal vascular endothelial cells by impairing the retinal microcirculation as with interferon therapy [11, 12]. Modification of leukocyte adhesion to the vascular endothelial cells and retinal capillary occlusion might have caused the vascular changes in our patient. 

The current case highlights the fact that ocular adverse events should be considered in patients treated with tocilizumab and suggests that analyses to detect infection and vascular changes are important for diagnosing and treating these conditions.

## Figures and Tables

**Table 1:** Laboratory data for the patient.

Property	Value
WBC	5970
Neutro	85.6%
Lymph	7.9%
Mono	5.9%
Eosino	0.6%
Baso	0%
RBC	3.47 × 10^6^ *μ*L
HGB	10.9 g/dL
PLT	239 × 10^3^ *μ*L
TP	7.1 g/dL
ALB	3.4 g/dL
BUN	10 mg/dL
Cr	0.42 mg/dL
Na	137 mEq/L
K	4.1 mEq/L
AST	23 U/L
ALT	22 U/L
LDH	229 U/L
CRP	0.88 mg/dL
C3	196 mg/dL
C4	14 mg/dL
IgG	1,840 mg/dL
RF	683 IU/mL

WBC: white blood cell; Neutro: neutrophil; Lymph: lymphocyte; Mono: monocyte; Eosino: eosinophil; Baso: basophil; RBC: red blood cell; HGB: hemoglobin; PLT: platelet; TP: total protein; ALB: albumin; BUN: blood urea nitrogen; Cr: creatinine; ALT: aspartate aminotransferase; ALT: alanine aminotransferase; LDH: lactate dehydrogenase; CRP: C-reactive protein; C3: complement 3; C4: complement 4, IgG: immunoglobulin; RF: rheumatoid factor.
